# The impact of digitization and conventional techniques on the fit of fixed partial dentures FPDs: systematic review and Meta-analysis

**DOI:** 10.1186/s12903-023-03628-1

**Published:** 2023-12-04

**Authors:** Esraa A. M. Saeed, Samar S. Alaghbari, Niu Lin

**Affiliations:** Prosthodontic Department, Hospital of Stomatology, Xian Jiaotong University, No. 98, West 5th Road, Zhonglou Commercial District, Xian City, 710003 Shaanxi China

**Keywords:** Marginal fit, Internal fit, Fixed partial dentures, Digital techniques, Meta-analysis

## Abstract

**Purpose of the study:**

The goal behind this study is to answer the question “In tooth-supported fixed partial dentures (FPDs), does the digital impression techniques compared to fabrications using conventional impression methods improve the marginal and internal fit?

**Background:**

The incorporation of digital technology in the fabrication of fixed partial dentures (FPDs) has accelerated over the past decade. This study is directed at evaluating the marginal and internal fit of FPDs manufactured using digital approaches compared to conventional techniques. The need for updated data has encouraged this review.

**Materials and methods:**

An electronic search was conducted in PubMed, Scopus, Web of Science, and the Grey Database to identify relevant studies. The Modified Methodological Index for Non-Randomized Studies (MINORS) was used to assess the risk of bias in in vitro experiments.

The key results of this meta-analysis were the standard mean differences (SMDs) and 95% confidence intervals (CI) of each main variance, marginal fit, and internal fit between the digital and conventional techniques.

Additional analyses were performed to assess the significance of three subgroup parameters: method of digitalization, cement spacer thickness, and span length, and their influence on the fit of the FPDs.

**Results:**

Based on predefined criteria, of the seven articles included in this systematic review, only five were selected for the quantitative data analysis. The marginal fit results were (*P* = 0.06; SMD: -1.88; 95% CI: − 3.88, 0.11) (*P* > 0.05) and the internal fit results were (*P* = 0.02; SMD: -0.80; 95% CI: − 1.49, − 0.10) (*P* < 0.05).

Regarding the subgroup analyses, the method of digitalization subgroup results were (*P* = 0.35; SMD: -1.89; 95% CI: − 3.89, 0.11) and (*P* = 0.80; SMD: -0.80; 95% CI: − 1.49, − 0.11) for marginal and internal fit, respectively. The span length results were (*P* = 0.10; SMD: -1.89; 95% CI: − 3.89, 0.11) for marginal fit and (*P* = 0.02; SMD: -0.80; 95% CI: − 1.49, − 0.11) for internal fit. The cement spacer thickness (*P* = 0.01; SMD: -1.89; 95% CI: − 3.89, 0.11) and (*P* = 0.04; SMD: -0.80; 95% CI: − 1.49, − 0.11) for marginal and internal fit, respectively.

**Conclusion:**

Tooth-retained fixed partial dentures FPDs produced by digital scanning and computer-aided design/computer-aided manufacturing (CAD/CAM) systems can significantly enhance the internal fit compared with those manufactured by traditional methods.

Intraoral scanners can replace conventional impressions for the fabrication of FPDs because they minimize the operating time and reduce patient pain.

Further clinical studies are required to obtain more conclusive results.

**Systematic review registration:**

This systematic review and meta-analysis was registered in the International Prospective Register of Systematic Reviews (PROSPERO), registration number CRD42021261397.

## Introduction

Dental impressions are commonly used to record oral structures in various dental fields [1]. An accurate impression is the most essential step in the construction of FPDs [2]. Inaccurate impressions may result in ill-fitting margins around the prostheses, plaque deposition, cement dissolving [3], and an elevated risk of pathogenic bacteria, which may also result in pulpal inflammation and necrosis. This causes abutment teeth to fail in various ways [4, 5].

The accuracy of dental impressions is critical for well-adapted restorations. The parameters that determine the fit of a dental restoration FPD are marginal and internal and contribute to its long-term duration [5, 6]. It refers to the degree of intimacy between the established abutment surface and the prostheses. The accuracy of dental impressions is critical for well-adapted restorations. Holmes et al. [7] defined the marginal gap (MG) as the vertical gap between the interior surface of the restoration and the margin of a prepared tooth, whereas the internal gap is the space from the same measurement to the axial wall. The marginal and internal fit of FPDs is determined by the size of the marginal and internal gaps [8].

Although the marginal discrepancy has various clinically acceptable values, McLean and von Fraunhofer presented a value of 120 μm as clinically acceptable as long as the internal fit is between 200 and 300 μm [9].

There are two methods of obtaining a dental impression, conventional impression technique and digital impression methods [10].

In conventional impression techniques, elastomers, such as polyether or polyvinyl siloxane (PVS), are commonly used to obtain impressions of prepared teeth because of their adequate precision and stability. Although conventional impressions have been the gold standard in the construction of multiple-unit fixed dental prostheses (MFDPs) for decades, inappropriate mold selection, material preparation, impression deformations before pouring, and stone model dimensional variations remain the most obvious drawbacks [10–12].

Digital dentistry has undergone dramatic improvements, and numerous CAD/CAM systems that induce intraoral scanning and dental prosthesis manufacturing have been widely accepted [13].

The CAD/CAM system includes two procedures [14]: the CAD process for data collection by digitalization with scanners and designing restorations using accurate software, and the CAM process for manufacturing restorations after data processing. Digitalization can be performed directly on the abutment tooth using an intraoral scanner or an extraoral/laboratory scanner on the impression or definitive model.

The most significant benefit of employing CAD/CAM over conventional methods is that it significantly reduces discomfort in patients who are often hesitant to make impressions using traditional techniques [15].

Additional advantages of employing CAD/CAM systems include technological advancements that have made it possible to minimize the shrinking process of the materials to be scanned while simultaneously improving patient convenience [15, 16]. Similarly, the geometry of the intraoral scanner’s light bulb has also been altered and reduced, making it more comfortable for its purpose, that is; the ratio of the apex bulb has been adjusted, allowing the scanner to detect all but the most demanding dental features with this system, notably the posterior teeth [17, 18]. The images acquired from the scan consume significantly less time than analog impressions [3], allowing the dental or technical team to identify errors and limitations at each step and correct them in CAD/CAM systems, where the scanner models, system software, and manufacturing machines are perfectly coordinated [19]. In addition, a digital mock-up provides the patient with an immediate future treatment plan and outcome [16].

Ultimately, this systematic review and meta-analysis aimed to determine whether digital scanning and manufacturing techniques may improve the marginal and internal fit of FPDs compared to conventional techniques. Furthermore, to assess the other variances that improve the marginal and internal fit of FPDs, as well as to evaluate whether intraoral scanners and CAD/CAM technology could be legitimate substitutes for traditional techniques in manufacturing FPDs.

The null hypothesis H0 states that, digital impression techniques produce FPD with similar marginal and internal fit compared to conventional techniques, while the alternative hypothesis H1, the digital impression technique could improve the marginal and internal fit of FPD compared to conventional methods.

## Materials and methods

### The research protocol and registration

**Fig. 1:**
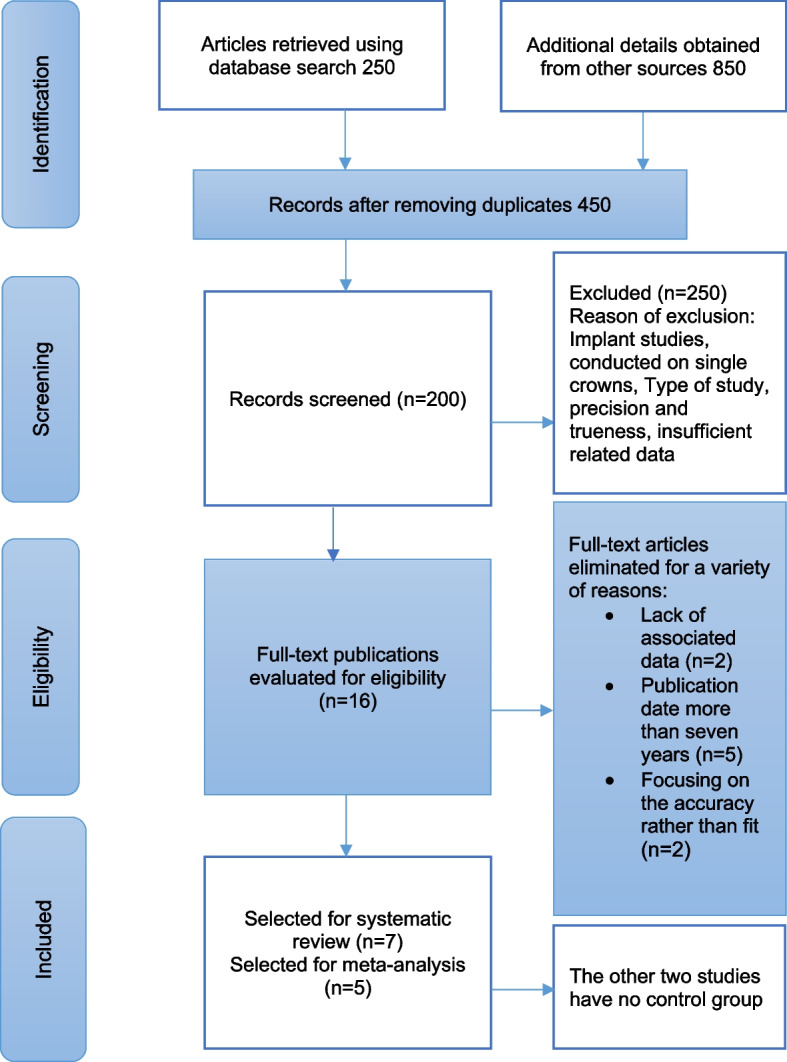
Flow chart of the selection process according to PRISMA guidelines

This systematic review and meta-analysis of in vitro studies followed the Preferred Reporting Items for Systematic Reviews and Meta-Analysis (PRISMA) guidelines. The study was registered in PROSPERO (Registration number: CRD42021261397). The full search strategy is illustrated in Fig. 1.

### The search strategy

Based on the Cochrane Handbook for Systematic Reviews of Interventions [16], the strategy passed through five major phases: formulation of the study question, search for relevant data, study eligibility, data extraction, and evaluation of the risk of bias.

### Formulation of the study question

Referring to the systematic review question formulation PICO, the questions for this study were as follows: “As for tooth-supported fixed partial dentures (FPDs) (P: population), does the digital impression techniques (I: intervention) compared to fabrications using conventional impression techniques(C: comparison) improve the marginal and internal fit (O: outcome)?”

The PICO question structure was also designed to solve two additional investigations regarding FPDs:

- Do the spacer thickness, span length, and digitalization methods affect the marginal and internal fit compared to traditional workflows?
- Does intraoral scanning technology replace conventional techniques for fabricating FPDs?

### Searching for relevant data

Multiple databases were used to conduct a rigorous search (PubMed, Scopus, and the Web of Science) and locate recently relevant published items until January 2022. In addition, grey literature was explored using Google Scholar. A special search was conducted on the annotated bibliographies of the selected studies. The search techniques and terms used in all the databases are illustrated in Table 1.

**Table 1:** The search protocol based on PICOS for each database and the associated finding

Database	Terms used and Search techniques	No. of results
PubMed	“Digital scanning or digital impression or dental scanning techniques or intraoral scanning technique or digital intraoral scanner impression or dental digitalization” AND “conventional impression or traditional impression techniques or analog impression or dental impression technique” AND “prostheses fit or marginal adaptation or marginal fit or marginal discrepancy or marginal misfits or marginal gap or marginal integrity” AND “internal fit or internal discrepancy or internal adaptation or internal gap or axial misfit or axial gap or axial discrepancy “AND “fixed dental prostheses or FDP or multiple units fixed partial dentures or MUFPD or fixed partial dentures or FPDs or fixed partial prosthesis”	180
Scopus	“Digital scanning or digital impression or dental scanning techniques or intraoral scanning technique or digital intraoral scanner impression or dental digitalization” AND “conventional impression or traditional impression techniques or analog impression or dental impression technique” AND “prostheses fit or marginal adaptation or marginal fit or marginal discrepancy or marginal misfits or marginal gap or marginal integrity” AND “internal fit or internal discrepancy or internal adaptation or internal gap or axial misfit or axial gap or axial discrepancy” AND “fixed dental prostheses or FDP or multiple units fixed partial dentures or MUFPD or fixed partial dentures or FPDs or fixed partial prosthesis”	70
Web of Science and Grey literature and other sources	“Digital scanning or digital impression or dental scanning techniques or intraoral scanning technique or digital intraoral scanner impression or dental digitalization” AND “conventional impression or traditional impression techniques or analog impression or dental impression technique” AND “prostheses fit or marginal adaptation or marginal fit or marginal discrepancy or marginal misfits or marginal gap or marginal integrity” AND “internal fit or internal discrepancy or internal adaptation or internal gap or axial misfit or axial gap or axial discrepancy” AND “fixed dental prostheses or FDP or multiple units fixed partial dentures or MUFPD or fixed partial dentures or FPDs or fixed partial prosthesis “	850

### Study selection criteria

#### Inclusion criteria

Experimental in vitro studies on tooth-supported FPDs using digital and conventional techniques. This review was open to papers published in any language, including English, Spanish, and Swedish.

The last 6 years of studies were only included in this article due to the tremendous improvements in digital technology. In addition, the rate of innovation in scanner systems and CAD/CAM technologies has progressed drastically over the last 5 years [20].

#### Exclusion criteria

Due to a lack of monitoring, sufficient data, and up-to-date information, we omitted in vivo randomized clinical trials and clinical research designs. Case studies, case series, descriptive studies, opinion articles, and cohort studies were also excluded. Similarly, studies based on scanning implant components or single restorations were also eliminated.

Before article screening and evaluation of the consistency and reliability of data collection, the reviewing process was conducted with the calibration of two reviewers (E.S and SSA) utilizing the inclusion criteria.

### Data extraction

The data from the included publications was collected in tables (Microsoft Excel 2016) and is illustrated in Table 2, to identify the key features of the chosen study: the author’s first name, publication year, specimen count per group (sample size), groups and impression techniques, unit count, and preparation type. The total internal gap was the average of all obtained values: cervical, axial, and occlusal gaps. The prosthetic marginal gap is defined as the mean of marginal, absolute marginal, vertical, and horizontal gaps [7].

**Table 2:** The retrieved data from the selected studies

Study author	Sample size	Groups	Sample size	Abutment teeth	Number units	Finish line
Shembesh et al. 2017	40	G 1: conventional impression G 2: model scan G 3: intra oral iTero scan G 4: intra oral Lava Definition	10 10 10 10	mandibular second premolar and mandibular right first molar	3-units	chamfer finish line
Kim et al. 2018	60	G1 DD: direct digitalization NP, P1, P2 G2 DI: indirect digitalization NP, 1P, 2P	30 30	premolar and molar First premolar and second molar	3-units 4-unit	chamfer finish line
Kocaağaoğlu et al. 2019	30	G1 Ci Conventional impression G2 Cdi cast scanning G3 Tdi digital impression	10 10 10	canine and second premolar teeth	3-units	shoulder finish line 1.0 mm in depth
Moustapha et al. 2019	30	Group C: conventional impression Group S: impression scan group Group T: intraoral scanner	10 10 10	central incisor and canine,	3-units	chamfer finish line
Arezoobakhsh et al. 2020	40	G1 CIL: Conventional impression G2 DCL: Dental Cast G3 TRI: TRIOS scanner G4 CSI: CS3600	10 10 10 10	maxillary first premolar and first molar	3-units	chamfer margin
Study author	Sample size	Group	Sample size	abutment	units	Finish line
Özal et al. 2021	48	G1: Trios 3 intra oral scanner G2: Trios 4 intra oral scanner G3: CEREC intra oral scanner G4: CEREC laboratory scanners: G5: InEos X5 G6: 3Shape D900L	12 12 12 12 12 12	maxillary left canine and second premolar teeth	3-units	deep shoulder marginal edge,
Uluc et al. 2022	60	3S-IOS, scanning mode 3S-IMP scanning impression 3S-STN scanning cast C-IOS, scanning model with Omnicam C-IMP, scanning impression with InEosX5 e C-STN,	10 10 10 10 10 10	Maxillary Right central, canine and second premolar	5-unit zirconia	chamfer diamond
Study author	Impression material used	Type of digital scanner	Definitive Restoration	Core material	Spacer thickness	Fabrication technique
Shembesh et al. 2017	One-step PVS impression technique	CadentiTero intraoral scanner Lava true definition (TT oxide powder) Extraoral scanners D700 laboratory scanner	zirconia FDPs	zirconia	35 μm cement space	Hard sintering with firing temperature at 1450°Cforabout 12 h
Kim et al. 2018	Two-stage impression PVS	Intraoral scanner: CEREC Omnicam; Extra oral scanners: inEos X5; Dentsply Sirona	Full zirconia FDP	Zirconia	Not mentioned	sintered to a furnace
Kocaağaoğlu et al. 2019	One-step with polyvinyl siloxane silicone	Intraoral scanners: CEREC Omnicom 3 shape TRIOS −3	LS framework	CoCr W	20 μm	Prototyping technology high precision high- energy laser
Moustapha et al. 2019	Two-stage impression PVS A.	3 shape TRIOS intraoral scanner lab scanner 1041imetric,	Full zirconia FDP	Zirconia	80 μm spacer	five-axis computer numerically controlled (CNC) machine
study author	Impression techniques	Type of the scanner	Definitive restoration	Material used	Spacer thickness	Fabrication techniques
Arezoobakhsh et al. 2020	Two-stage impression PVS	3 shape TRIOS intraoral scanner CS3600 intraoral scanner	LS framework	zirconia	35 μm	5-axis milling machine (Versamill 5X200; Axsys Dental Solutions)
Özal et al. 2021	2-step putty/wash polyvinyl siloxane	3Shape Trios 3 (T3) 3Shape Trios 4 (T4) CEREC AC Omnicam CEREC Primescan Laboratory scanners: CEREC InEos X5 3Shape D900L (3Shape)	Full zirconia FDP	Zirconia	50 μm	milling device (milling axes). sintered inFire HTC for 90 min at 1500 °C
Uluc et al. 2022	Scannable polyvinyl siloxane (PVS) impression material	Trios 3 (3Shape Omnicam SironaDental E3, 3Shape, Cerec, InEosX5, Sirona Dental	Full zirconia FDP	Zirconia	50 μm.	Redon Hybrid Technology, 5-axis milling machine
Study author	Method of assessing the gap	Number of measuring points	Marginal, Mean ± SD	Internal gap, Mean ± SD		
Shembesh et al. 2017	Optical comparator (Horizontal Optical Comparator)	mesial, distal, buccal, lingual	group 1: 81.4 μm ± 6.8 group 2: 50.2 μm ± 6.1 group3:62.4 μm ±5.0 group 4:26.6 μm ± 4.7			
Kim et al. 2018	Replica technique at ×50 magnification	5 measurements were obtained from the buccal colingual sectioned planes: buccal-MG, buccal-AG, OG, lingual-AG, and lingual-MG.	Group DD MG: marginal gap: NP 61 ± 13 1P 62.36 ± 10.3 2P 69.38 ± 13.55 NP 108.93 ± 19.74 1P 110.42 ± 18.47 2P 127.07 ± 20.47	CIL: 238 ± 92 DCL:248 ± 71 TRI: 104 ± 27 CSI: 128 ± 16		
Kocaağaoğlu et al. 2019	Stereomicroscope 30 × magnification	Marginal discrepancy in 4 points: Mesial distal buccal and palatal	Ci: 98.8 ± 16.43 Cdi 63.78 ± 14.05 Tdi: 65.14 ± 18.05			
Moustapha et al. 2019	Replica technique magnification of 260×	marginal Chamfer Axial incisal	C 30 ± 10 S 27 ± 7 T 20 ± 5	C: 84 ± 11 S: 78 ± 9 T: 70 ± 11		
Study author	Method of assessing the gap	Number of measuring points		Marginal and internal gap, Mean ± SD		
Arezoobakhsh et al. 2020	The replica technique stereomicroscope Microsystems) at × 50 m	Marginal, axial, occlusal gap		Marginal discrepancy: CIL: 91 ± 40 DCL:106 ± 45 TRI:60 ± 15 CSI:55 ± 13	Internal discrepancy/gap: CIL: 238 ± 92 DCL:248 ± 71 TRI: 104 ± 27 CSI: 128 ± 16	
Özal et al. 2021	Silicone replica method at ×40 magnification.	4 points: Marginal axial, axio-occlusal occlusal		Marginal discrepancy: G1: 98.9 ± 22.3 98.1 ± 16.8 G2: 97.6 ± 26.6,92.8 ± 12.4 G3: 91.6 ± 19.1, 105.3 ± 18.5 G4: 85.4 ± 12.0, 86.9 ± 19.2 G5: 114.4 ± 14.1, 105.3 ± 11.2 G6: 128.4 ± 10.9, 139.5 ± 16.0	Internal discrepancy/gap: 93.8 ± 26.9 98.4 ± 16.8 93.3 ± 16.2 92.5 ± 11.10 105.4 ± 13.7113.4 ± 17.4 106.4 ± 8.2114.5 ± 12.0113.3 ± 16.9122.7 ± 13.7 118.2 ± 8.5132.1 ± 6.10	
Uluc et al. 2022	Silicone replica method inspection and metrology (overlapping) method	1900 points on each prepared tooth: Marginal Occlusal Axial		Marginal discrepancy: 3SIOS 76.2 ± 10 3S-IMP 81.7 ± 8 3S-STN 82.6 ± 8 C-IOS 80.3 ± 6	Internal discrepancy/gap: 3S-IOS 80.1 ± 10 3S-IMP 86.9 ± 13 3S-STN 86.6 ± 10 C-IOS 75.3 ± 10 C-IMP 80.3 ± 15	

### The methodological quality and risk of bias assessment MINORS

This tool (MINORS) is commonly used to evaluate the risk of bias in in vitro studies. It consisted of 11 items. The criteria were scored as follows: 2 if data were available and adequate, 1 if data were not adequately reported, and 0 when data are unavailable. Table 3 illustrate the scores for each included study.

**Table 3:** Scores obtained for each included study using MINORS

Evaluation items	Shembesh	Kim	Kaggolu	Moustapha	A.A	Özal	Uluc
1-basically stated purpose	2	2	2	2	2	2	2
2-Contemporary groups	2	0	2	2	2	0	2
3- Scanning technique based on guideline	2	2	2	2	2	2	2
4-Control groups	2	1	2	1	2	0	1
5-Definitive restoration	1	0	0	1	2	2	2
6-Blindness of observer or statistician	2	2	0	1	0	1	0
7-Sufficient number of observations in every study	1	2	1	2	0	1	1
8-Sufficient method of observation to assess the gap	2	2	2	2	2	2	2
9- Standard technique for tooth preparation	2	2	2	2	2	2	2
10-Statistical analysis.	2	2	2	2	2	2	2
SCORE	18	15	15	17	16	14	16

Total score: equal to or greater than 18, it indicated a low risk of bias; if equal to or more than 16, it indicated a moderate risk; and if it was equal to 15 or less, it indicated a high risk of bias

### Data analysis

The analysis was carried out on 200 FPDs: 60 were constructed using conventional methods and 140 via digital techniques (of which 60 models were scanned with an intraoral scanner and 80 models were scanned with a laboratory scanner). The key result measurement in this study was the SMD of each of the two variables, marginal and internal fit, constructed using two impression techniques, digital and conventional, utilizing the following formula:

$$\frac{\text{Mean gap in the digital techniques - Mean gap in the conventional techniques}}{\text{Pooled standard deviation}}$$

The internal fit was categorized as the mean of all available internal gap values illustrated in the studies: axial, cervical, and occlusal gaps.

The secondary results evaluated the effect of the digitalization method, span length, and cement space thickness on the marginal and internal fit.

The quantitative analysis was calculated from the mean with a 95% confidence interval for each effect size of each subgroup, depending on the SMD [21]. All statistical analyses were performed using the statistical program STATA.

## Results

### Search results

The electronic search identified 1100 articles, 180 from PubMed/MEDLINE, 70 from Scopus, and 850 from other sources (Web of Science, Google Scholar, and Grey Database). After duplicate articles had been removed, 250 articles were excluded during the first screening stage for several reasons. Some experiments were conducted on single restorations and implants, and others were in vivo and case report studies.

In the second stage, accompanying the eligibility criteria, screening of titles and abstracts of articles resulted in 16 articles; of these, seven studies were included in the systematic review, and the remaining were omitted as follows: five studies were published more than 7 years ago, two studies lacked related data, and the last two focused mainly on accuracy and precision rather than fit or adaptation. In the last search phase, seven full texts were comprehensively screened, and only five experiments were included in the meta-analysis, as two studies have yet to reveal a control group. The full selection process according to the PRISMA guidelines is illustrated in Fig. 1.

### Results from the extracted data in the included studies

The chamfer margin was prepared in most studies, whereas the shoulder margin was used in two studies. In addition, three dissimilar gap measuring techniques were used: the replica technique with stereomicroscope was the predominant method in assessing the marginal and internal gaps; the other techniques include the optical comparator and scanning electron microscopy.

Among all available scanners, six intraoral scanners were utilized in the included studies: Lava True Definition, iTero, TRIOS 3S, TRIOS 4S, Cerec Omnicam, and Primescan. Since diverse oral scanners employ various image collection technologies, their scanning precision and accuracy vary significantly.

### The methodological quality and risk of bias assessment

The overall assessment results of each study are presented in Table 3. All studies stated a clear aim for the study objectives, scanning techniques according to guidelines, sufficient methods to assess the gap, tooth preparation technique, and statistical analysis.

In contrast, except for a single study [19], which had a low possibility of bias, all studies demonstrated a moderate to high risk of bias in terms of the blindness of the observer and the adequate number of observations. Three studies had total scores of 17, 16, and 16, respectively, indicating a moderate risk of bias whereas three studies scored 15, 14, and 14, respectively, indicating a greater possibility of bias.

### Meta-analysis results

Of the seven studies included in this systematic review, only five were eligible for meta-analysis. Two studies were excluded as they lacked a control group. Five studies were used to compare the marginal fit, and three experiments were used to compare the internal fit between the two impression techniques.

All analyses were measured as means with a 95% confidence interval for each size model of each group, depending on the SMD.

I^2^ tests demonstrated 96.69% in the marginal fit and 65.93% in the internal fit, revealing a significant heterogeneity between the included studies.

#### Marginal fit results

**Fig. 2:**
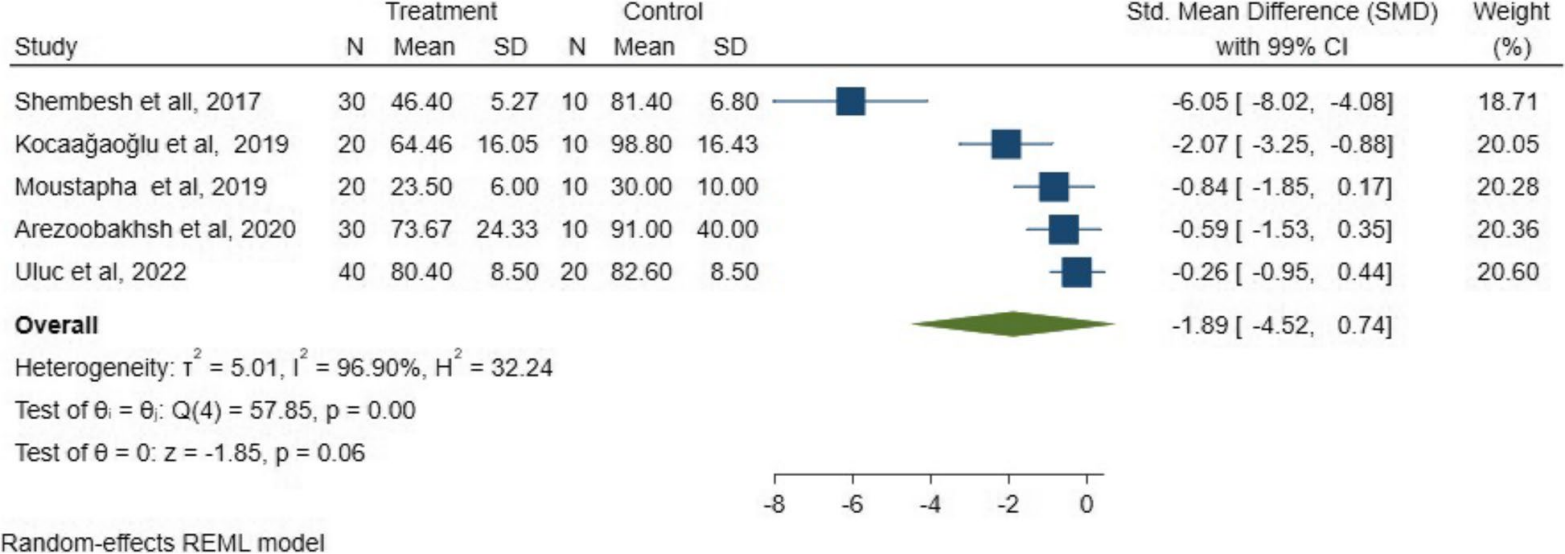
SMDs with a 95% confidence interval in the marginal fit between digital and conventional techniques among the included studies and overall results

The assessment was conducted to evaluate the marginal fit, and the results of the meta-analysis are shown in Fig. 2. Based on the assumption that the outcomes of the included studies preferred the digital approach, the marginal fit results revealed a statistically non-significant difference between digital and conventional workflows (*P* = 0.06; SMD: -1.88; 95% CI: − 3.88, 0.11) (*P* > 0.05).

**Fig. 3:**
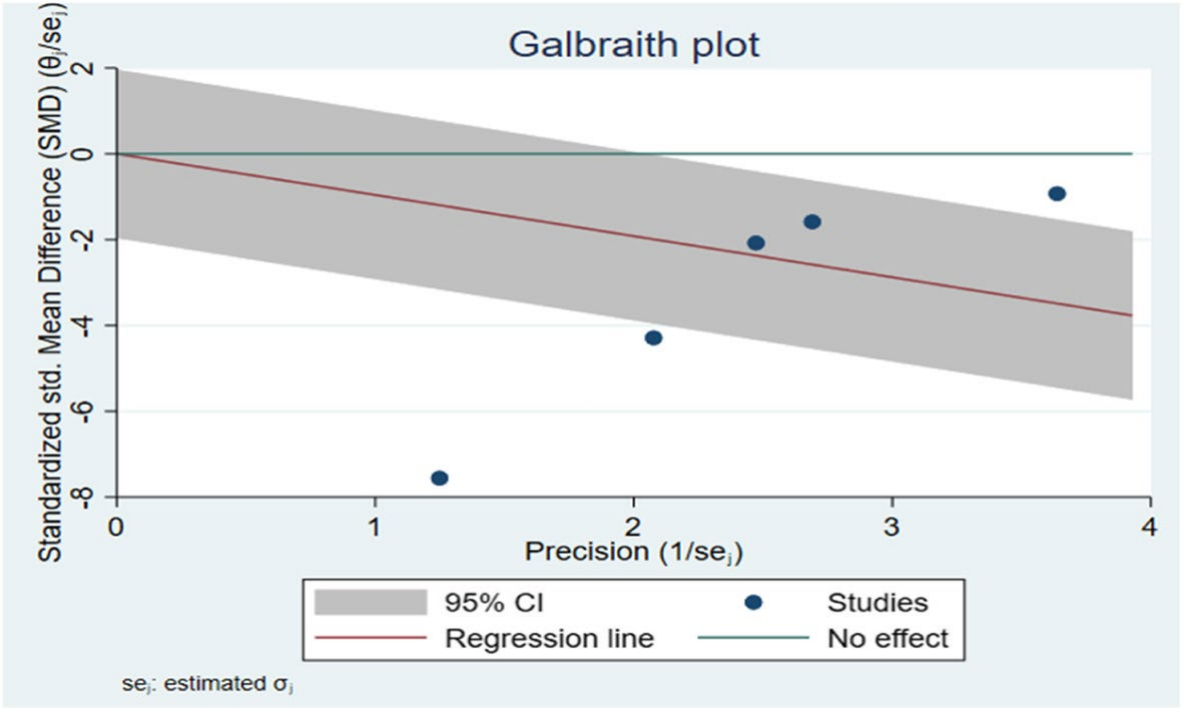
The Galbraith plot and meta-regression in the included studies indicated a risk of bias

**Fig. 4:**
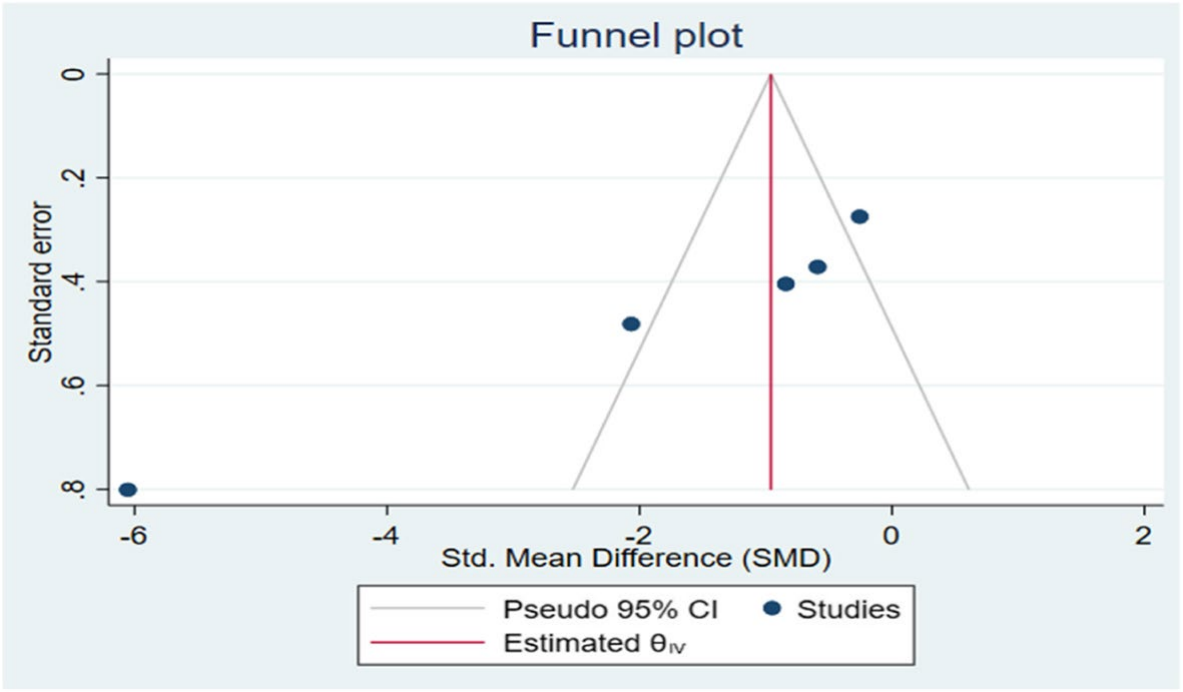
The funnel plot with Pseudo 95% confidence intervals in the marginal fit among the studies

**Fig. 5:**
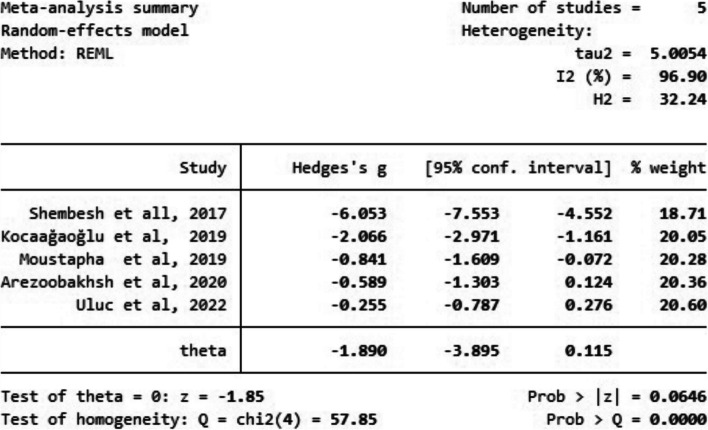
Effect size models of the included studies

Galbraith chart Fig. 3 and Funnel plot Fig. 4 were used to show the distribution of effect values to assess the possibility of publication bias. The random-effects analysis was further done, and showed no association between SMD and the small size effect Fig. 5.

Subgroups analyses:

Further analyses were conducted to assess the effects of each influencing factor on marginal fit, the digitalization method, span length (number of units), and cement space thickness.

**Fig. 6:**
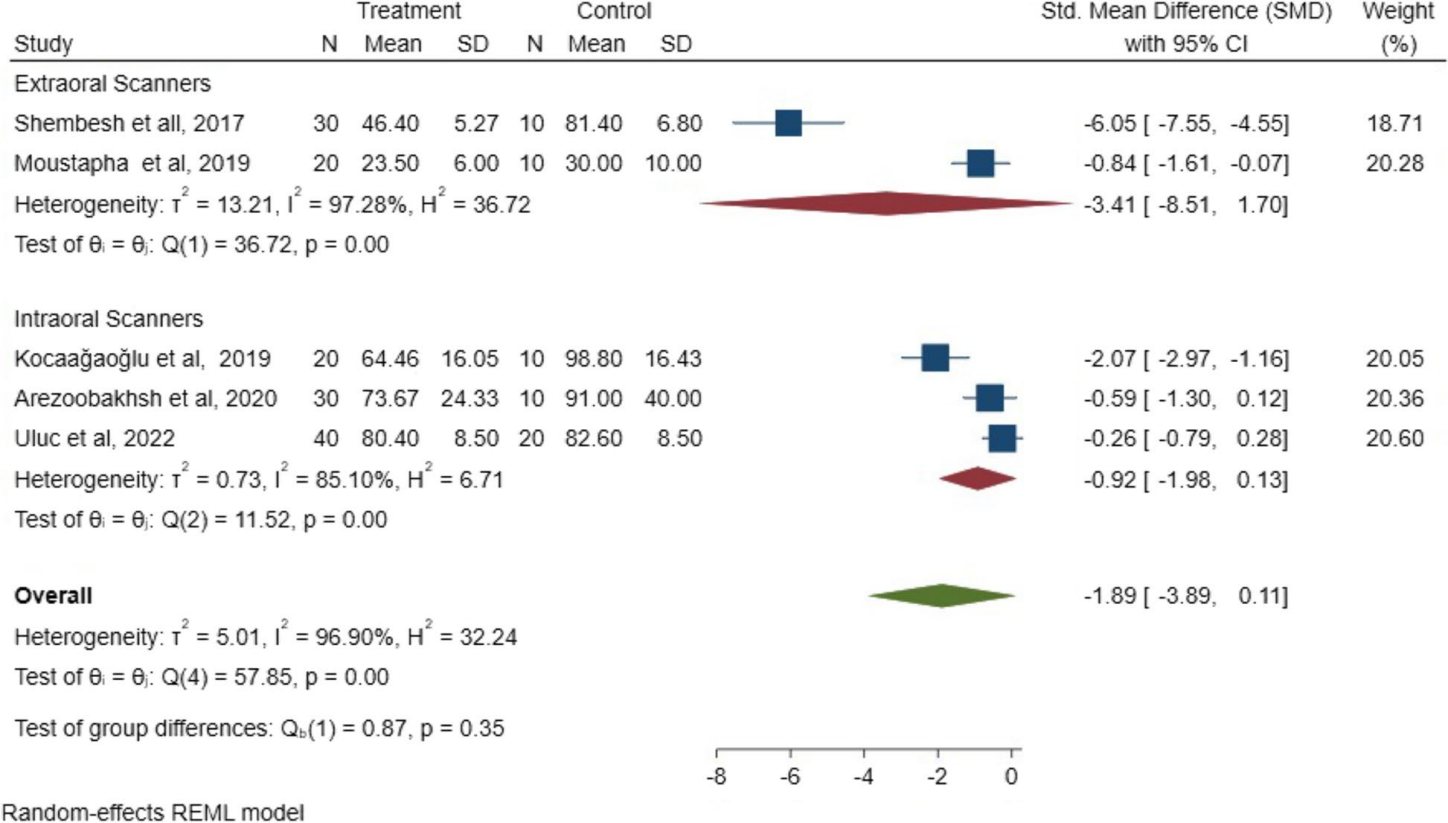
SMD of the marginal fit between the direct and indirect scanning groups

Subgroup 1:

The analysis investigated the influence of direct and indirect digitalization on the marginal fit. Although the results of the included studies favored the full digital approach over partial techniques, the results showed a non-significant difference between the direct and indirect scanning (*P* = 0.35; SMD: -1.89; 95% CI: − 3.89, 0.11) (*P* > 0.05). The SMD results are illustrated in Fig. 6.

**Fig. 7:**
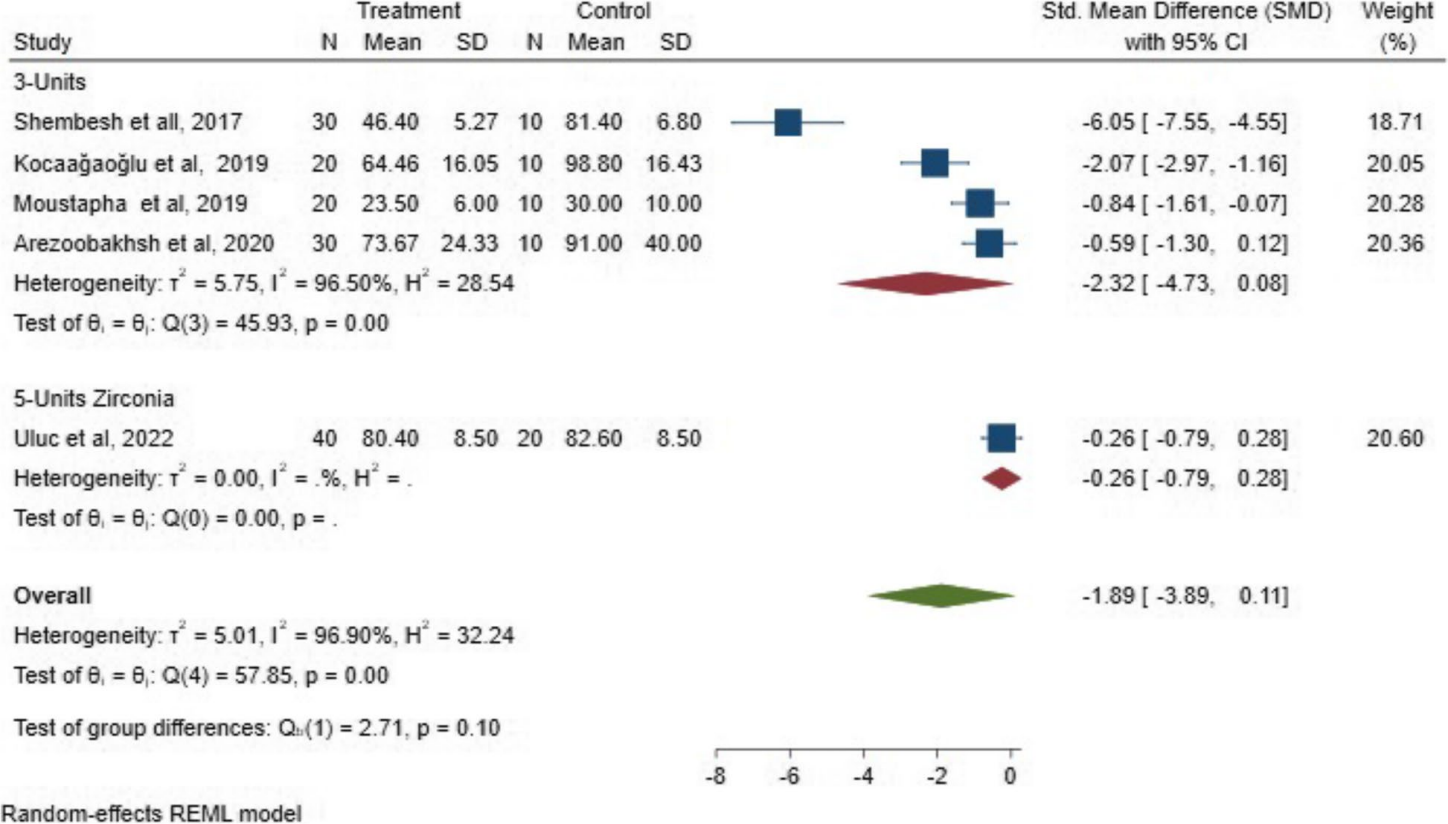
The SMD of the span length on the marginal fit between 3- and 5-unit FPDs

Subgroup 2:

Regarding the span length evaluation, the analysis revealed a statistically non-significant difference in the marginal fit for three- and five-unit FPDs (*P* = 0.10; SMD: -1.89; 95% CI: − 3.89, 0.11) (*P* > 0.05). The SMD results of the span length in marginal fit are illustrated in Fig. 7.

**Fig. 8:**
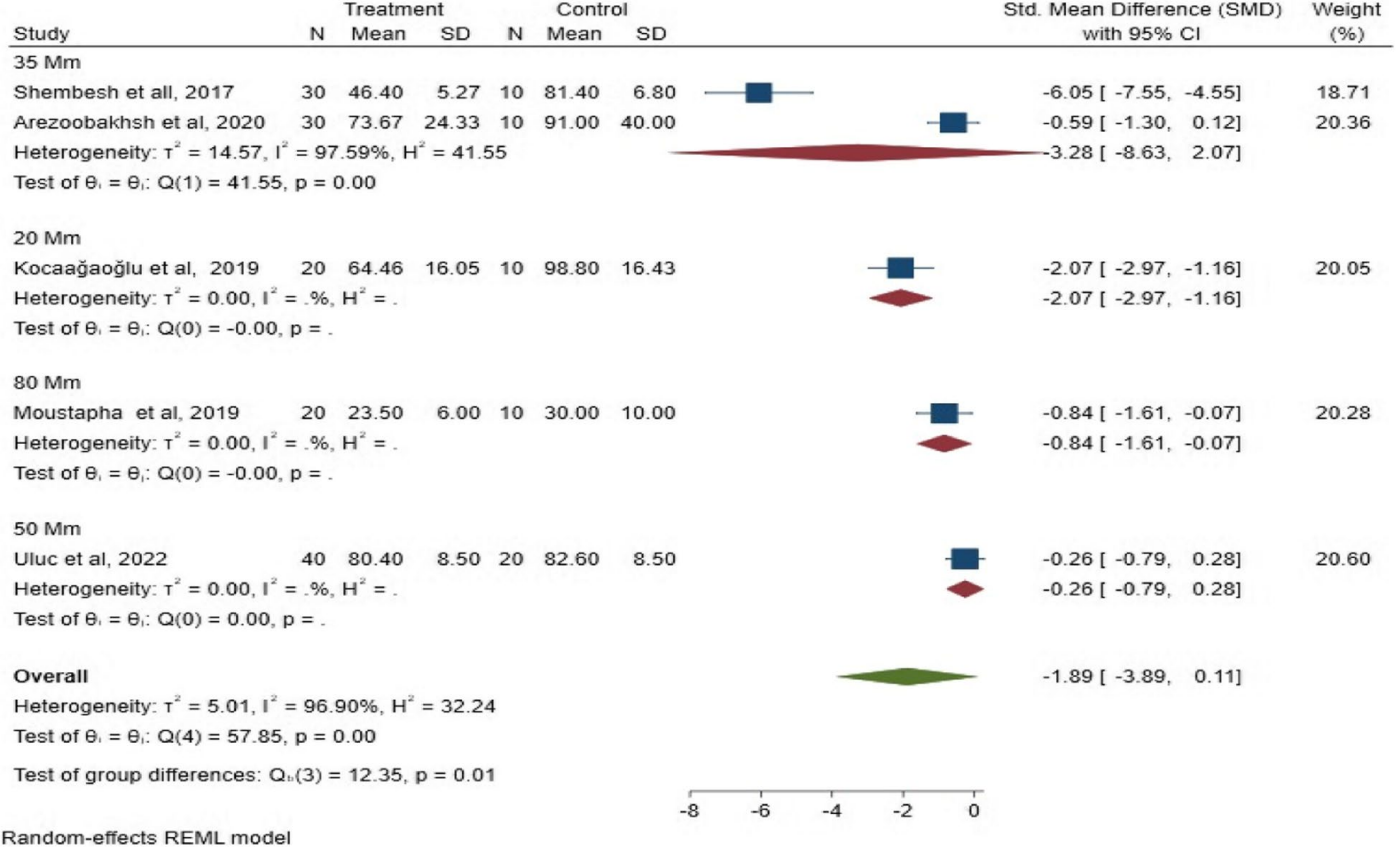
The SMD of cement space thickness on the marginal fit

Subgroup 3:

The analysis was performed on the thickness of the cement space. The results showed a statistical difference between the studies in the marginal fit when using different spacer thicknesses; 35 μm, 20 μm, 80 μm, and 50 μm (*P* = 0.01; SMD: -1.89; 95% CI: − 3.89, 0.11) (*P* < 0.05). The SMD results of cement space thickness on the marginal fit are illustrated in Fig. 8.

#### Internal fit results

**Fig. 9:**
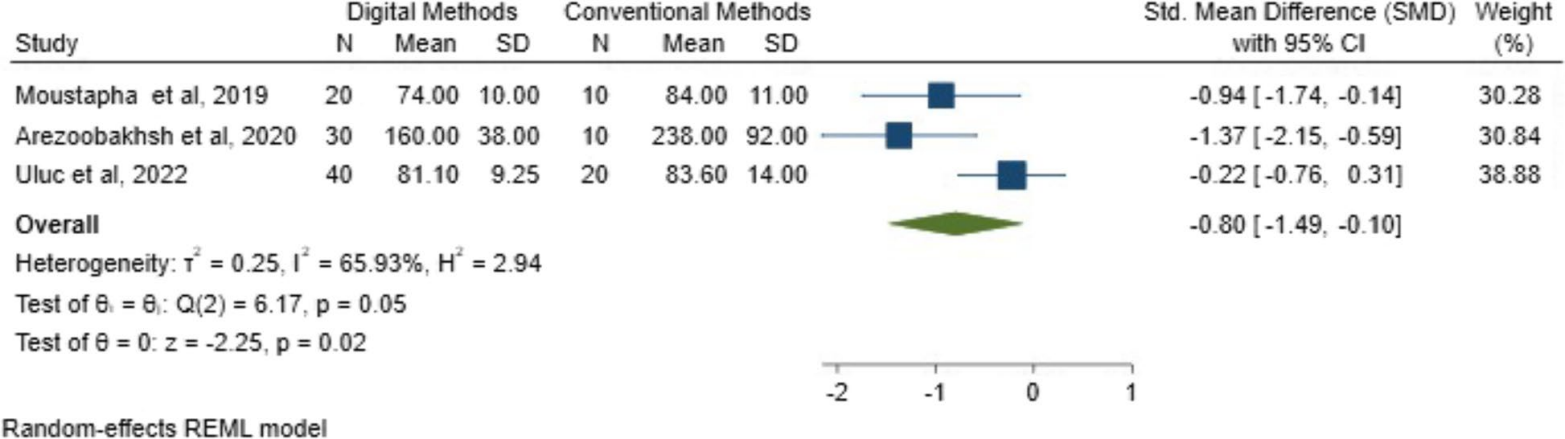
SMDs with 95% confidence interval of the internal fit between digital and conventional techniques among the included studies and overall results

The mean and SMDs for the intaglio fit between digital and conventional workflows are illustrated in Fig. 9. The analysis indicated a statistically significant difference in the internal fit between digital and conventional workflows (*P* = 0.02; SMD: -0.80; 95% CI: − 1.49, − 0.10) (*P* < 0.05).

**Fig. 10:**
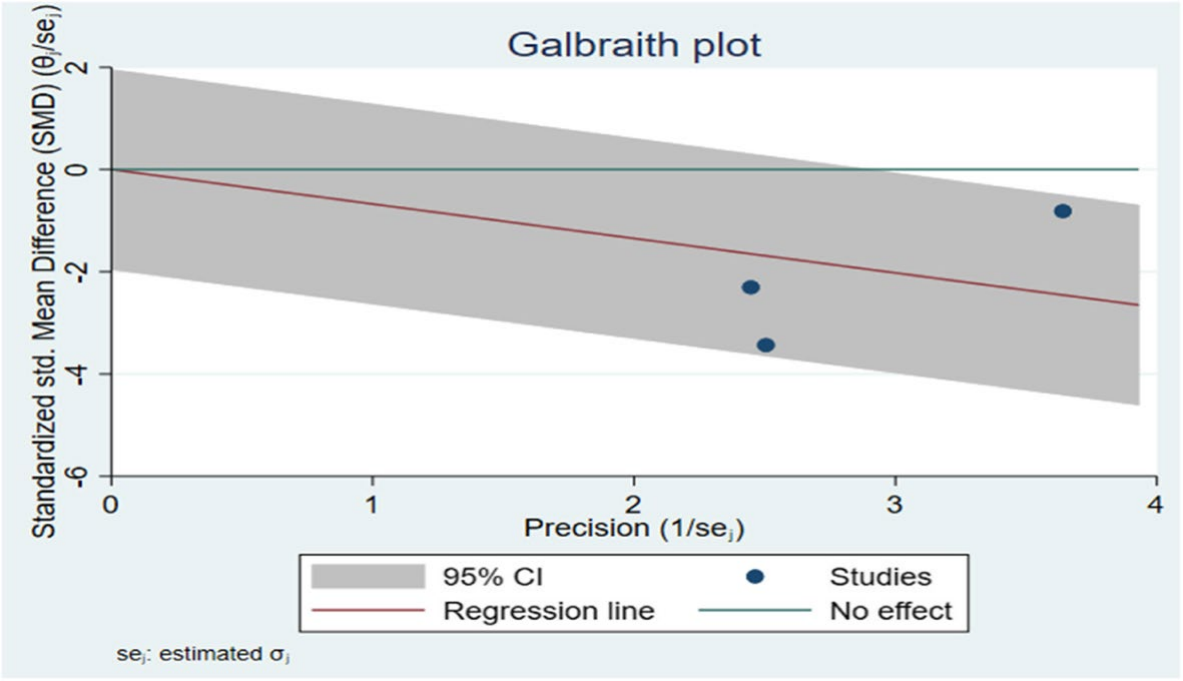
The Galbraith plot and meta-regression in the included studies

**Fig. 11:**
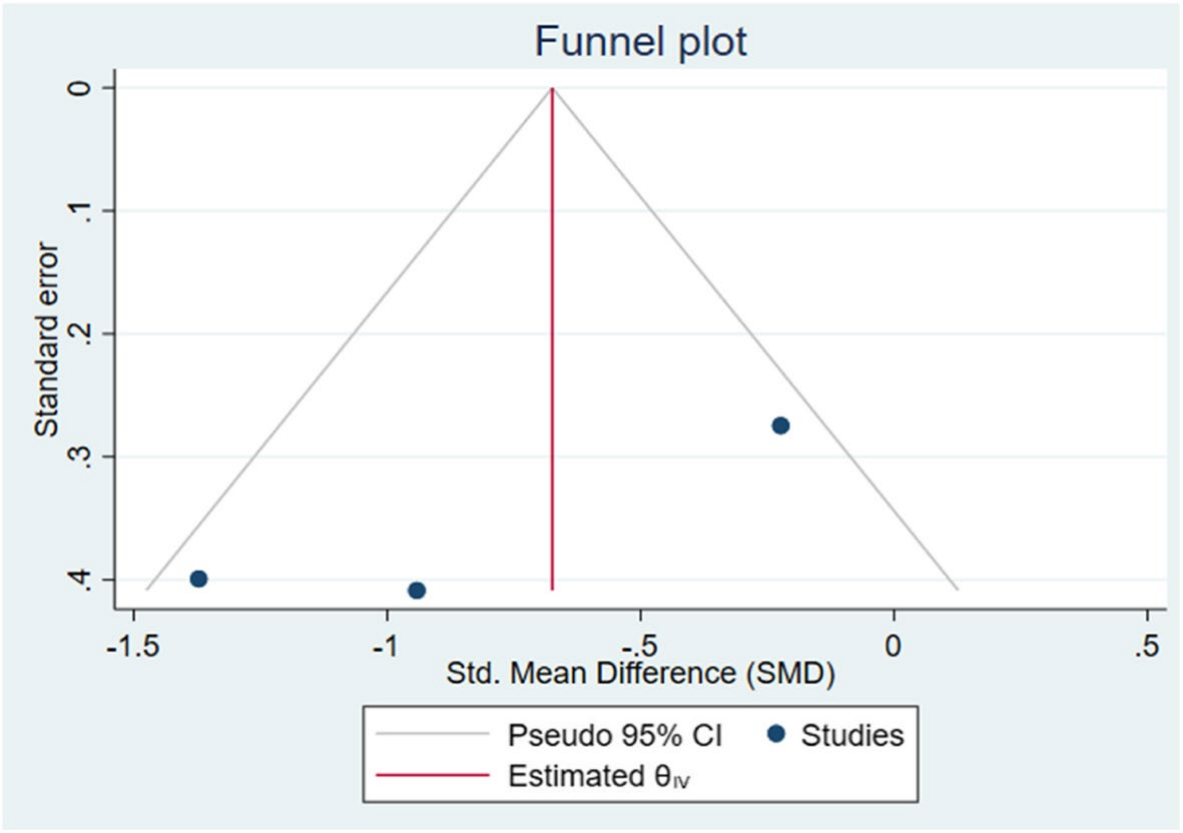
The funnel plot with Pseudo 95% confidence intervals for internal fit among the studies

**Fig. 12:**
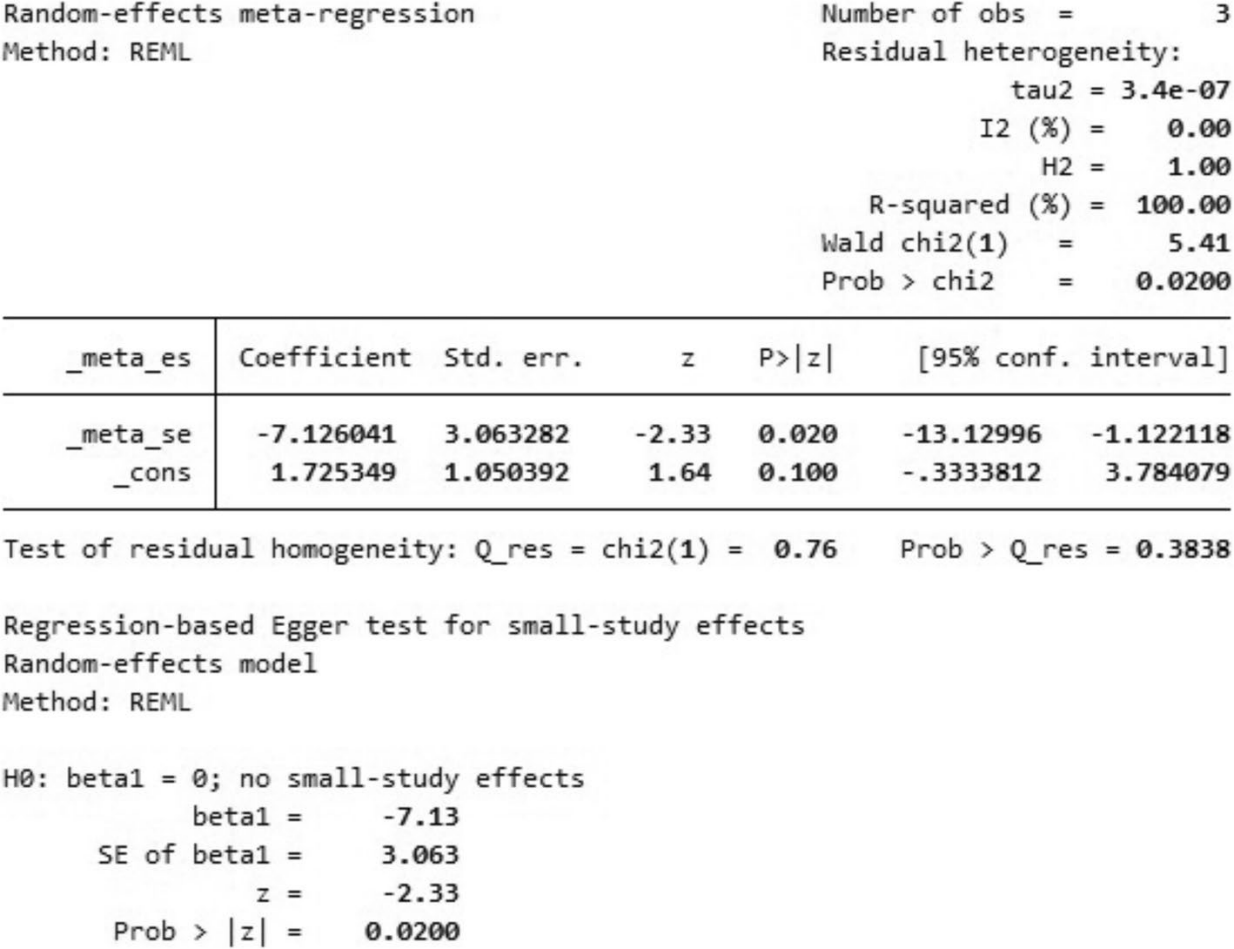
Effect size models of the included studies

Galbraith chart Fig. 10 and Funnel plot Fig. 11 were used to show the distribution of effect values to assess the possibility of publication bias. The random-effects analysis was further done, and showed an association between SMD and the small size effect Fig. 12.

**Fig. 13:**
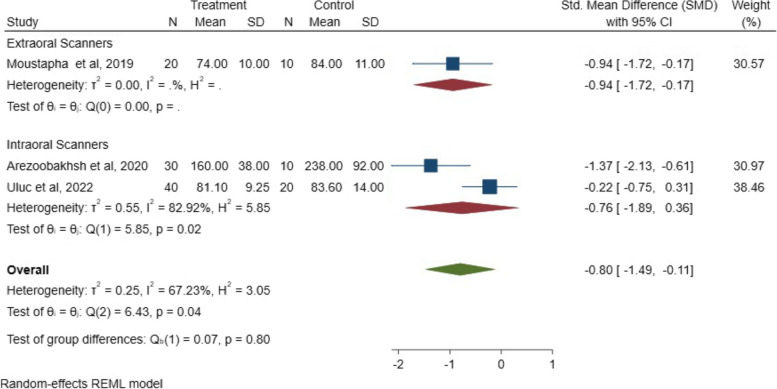
Internal fit SMD results between direct and indirect digitalization methods

Subgroups analyses:

- Subgroup 1:

This analysis focuses on the impact of the digitalization approach on internal fit. Although the results presented in the included studies favored the full digital approach rather than partial techniques, the difference in the internal fit between the extraoral and intraoral scanning groups is statistically insignificant, (*P* = 0.80; SMD: -0.80; 95% CI: − 1.49, − 0.11) (*P* > 0.05). The SMD results between direct and indirect digitalization methods are presented in Fig. 13.

**Fig. 14:**
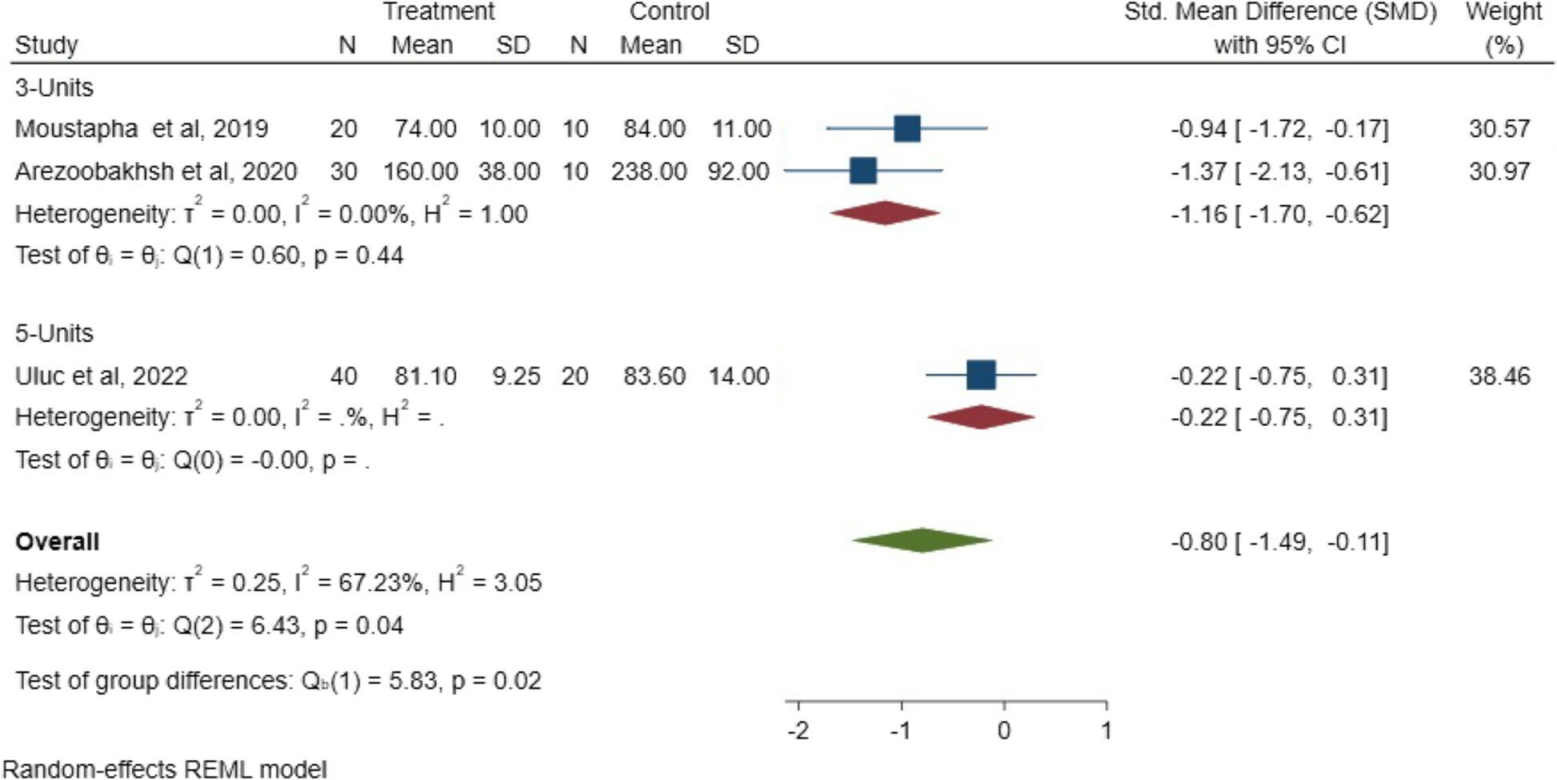
The SMD of the span length on the internal fit between 3- and 5-unit FPDs

- Subgroup 2:

In the span length analysis, there was a significant difference in the internal fit between the two groups for three-unit and five-unit FPD (*P* = 0.02; SMD: -0.80; 95% CI: − 1.49, − 0.11) (*P* < 0.05). This indicates that the span length/number of units can inversely affect the internal fit of FPD. The SMD results of the span length on the internal fit between 3- and 5-unit FPDs are illustrated in Fig. 14.

**Fig. 15:**
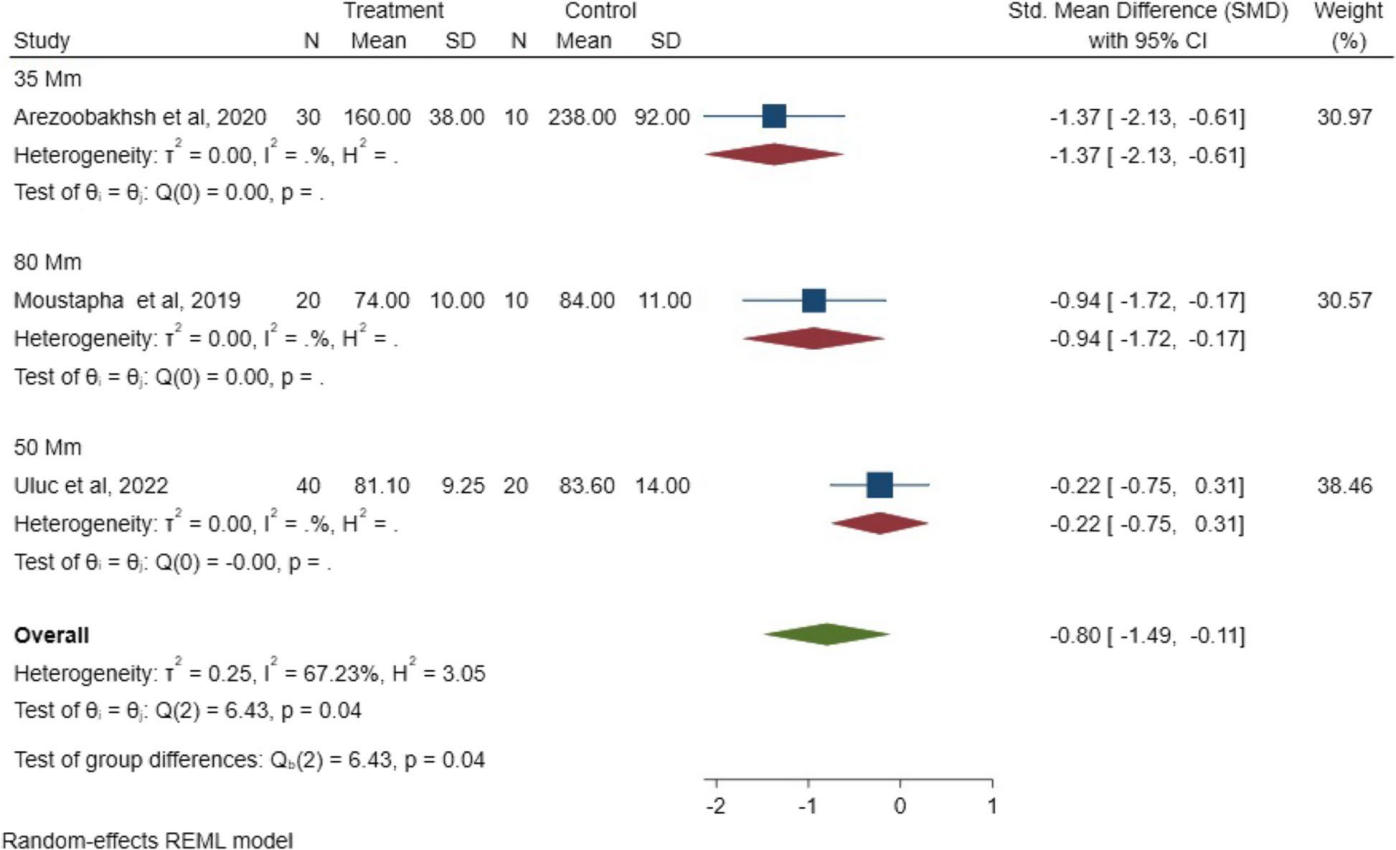
The SMD of space thickness on internal fit

- Subgroup 3 analysis:

Regarding the cement space thickness, a significant statistical difference (*P* = 0.04; SMD: -0.80; 95% CI: − 1.49, − 0.11) (*P* < 0.05) was observed between the three- and five-unit FPDs, indicating that spacer thickness inversely affects the internal fit of FPDs. The SMD results of cement space thickness on the internal fit are illustrated in Fig. 15.

## Discussion

Recent data regarding the fit of tooth-supported FPDs that compare digital and conventional workflows are contradictory and heterogeneous, the need for updated results has encouraged this review. According to the relevant literature, this study will be the first review of scientific research discussing FPDs’ marginal and internal fit fabricated by digital and traditional approaches, with a focus on published studies within the last 6 years. The year 2017 was chosen as the cutoff for study inclusion because the rate of innovation in scanner systems and CAD/CAM technologies has drastically progressed over the last 5 years. The earliest studies revealed the greatest mean difference between digital and traditional approaches, which may have affected the analysis results [20].

Clinical studies were excluded as there were few published in vitro experiments and even fewer in vivo studies evaluating the fit of FPDs in terms of marginal and internal fit using digital and conventional workflows [22]. Ethical concerns aside, intraoral environmental challenges that restrict the scanning procedure, the swallowing movements, the existence of blood or saliva, and involuntary tongue movement may jeopardize the digitalization procedure [18]. Additionally, the results were variable, and the majority of studies confirmed the presence of many confounding factors that may have affected the analysis results. Moreover, no study has evaluated the survival and follow-up of full-coverage restorations or fixed partial dentures [23]. Furthermore, two recent meta-analyses were based on in vivo studies, obviating the need for a second analysis [22, 24].

The outcome of this analysis indicated that tooth-supported FPDs fabricated by digital techniques significantly enhance the internal fit but it didn’t influence the marginal fit compared to fabrications using conventional methods.

These results are compared to a meta-analysis by Russo et al. [25], a greater marginal gap value was observed in MFDPs fabricated by scanning systems than in those fabricated by conventional techniques, however, the difference was not statistically significant. In contrast, Morsy et al. [26] revealed that the marginal and internal fit of FPDs were significantly enhanced by digital scanning. In this study, a single clinical study and eight experiments were selected for the meta-analysis. Nevertheless, the clinical and experimental values may conflict with and mislead the outcome. Furthermore, the analysis did not fully evaluate the direct and indirect scanning technique results, and the publication period of the included studies is another factor that could influence the results of this analysis.

Regarding the method of digitalization, the results using extraoral and intraoral scanners showed no significant difference in marginal and internal fit, however, intraoral scanners could replace conventional impressions for the fabrication of FPDs because they minimize the operating time and remove patient pain. They also reduce fabrication processes, which may lead to errors due to less coordination between the clinic and dental laboratory.

Three different fabrication methods were observed in some of the included studies, and the results were comparable to those of different combinations of the fully conventional method, full digitalization, and partially digital groups. Conventional impressions and/or stone casts were scanned using an extraoral scanner. Even though the included studies preferred intraoral scanners over conventional or partial techniques, the statistical difference was insignificant.

A comprehensive review of intraoral scanner precision has been performed [18]. For short-span FPD impressions, the accuracy of the IOS was still similar to that of PVS and polyether impressions. As the span increased, the accuracy of the impression of the PVS became evident. Hasanzade et al. [21] observed that a fully digital workflow is superior to conventional techniques in terms of marginal fit. The authors suggested that the majority of inconsistencies in conventional or partial workflows were induced by stone-cast fabrication. However, in a fully digitalized group, the scanner systems, design software, and milling machines are appropriately surpassed, and the errors in each process can be corrected.

When evaluating the effect of the span length /number of units based on the outcome, digital techniques can significantly enhance the internal fit of three and five-unit FPD, however the difference in the marginal fit between the digital and conventional workflows for three-unit and five-unit FPDs is statistically non-significant. Another study revealed that marginal and internal fit were significantly affected by the edentulous span length of three- and four-unit fixed partial dentures, the study also discovered that the digitalization system produces fewer marginal and internal discrepancies than traditional techniques in up to 4-unit zirconia FPDs [21]. This significant difference is probably due to the marginal and internal gap values in the experimental findings in early published literature (2017–2012).

Regarding the cement spacer thickness, the results revealed a significantly better marginal and internal fit with digital workflow than a conventional workflow. The internal gap of the three-unit FPDs was significantly smaller when the spacer thickness decreased [26].

As the number of clinical studies is limited, the clinical significance of this study is that the digital scanning technique is a developing technology, and it is essential to test in standard situations while eliminating confounding factors. The results of this study will help make an initial judgment about the superiority of digital and conventional methods before making a conclusive decision about their clinical performance.

There are many reasons could explain the heterogeneity between the studies; the small number of included studies, increased bias in most of the selected studies, and experimental differences such as dissimilar scanner models utilized among experiments, study sample size, impression materials, preparation design, measuring method, fabrication machines and techniques, milling system, and data analysis tests used in each study.

The possible source of bias in marginal fit results between the digital and conventional group is mainly due to methodological difference between the included studies, however in internal fit analysis a publication bias was noticed due to small effect size.

## Conclusion

This systematic review and meta-analysis produced specific conclusions based on these findings:

1. The study revealed that tooth-supported FPDs manufactured using CAD/CAM technology significantly improved the internal fit, but did not affect the marginal fit.
2. Although the results of digitalization methods using extraoral and intraoral scanners showed no significant difference in marginal and internal fit, intraoral scanners could replace conventional impressions for the fabrication of FPDs because they minimize the operating time and remove patient pain. They also reduce fabrication processes, which may lead to errors owing to less coordination between the clinic and dental laboratory.
3. Digital procedures exhibited a significant difference in internal fit between three- and five-unit FPDs, and lengthening the span of FPDs negatively impacted their fit.
4. The thickness of the cement space inversely influences the marginal and internal fit of the FPDs.

The results should be interpreted cautiously, as they were conducted on a limited number of studies throughout a limited period. Besides, the findings focused mostly on experiments conducted in laboratory settings.

Fewer clinical studies in the published literature mean less conclusive results; thus, more updated clinical studies with success and survival rates are needed to provide a stronger evidence.

## Data Availability

An electronic search was conducted on different databases including PubMed, Scopus, Web of Science, and Grey literature to uncover relevant studies. The datasets generated and analysed during the current study are available in the Raw data excel sheet https://1drv.ms/x/s!AsFQHSyHP8BRgwqL66MFzmLrIcgF.

## Abbreviations

FPDs: Fixed Partial Dentures
MINORS: The Modified Methodological Index for Non-Randomized Studies
SMDs: standard mean differences
CI: Confidence Interval
CAD-CAM: Computer-aided design/Computer-aided manufacturing
IOS: Intraoral scanner
PROSPERO: Preferred Reporting Item for Systematic Review and Meta-Analysis
MG: Marginal gap
PVS: Polyvinyl siloxane
MFDP: Multiple-unit fixed dental prostheses
PRISMA: Preferred Reporting Items for Systematic Review
